# Reply

**DOI:** 10.1002/hep4.2084

**Published:** 2022-08-26

**Authors:** Yuanqing Lu, Liqun R. Wang, Jungnam Lee, Naweed S. Mohammad, Alek M. Aranyos, Calvin Gould, Nazli Khodayari, Regina A. Oshins, Craig G. Moneypenny, Mark L. Brantly

**Affiliations:** ^1^ Division of Pulmonary, Critical Care and Sleep Medicine, Department of Medicine University of Florida Gainesville Florida USA

We agree that the mechanisms of up‐regulation of C/EBP homologous protein (CHOP) in this model remain unclear.^[^ ^1^, ^2^ ^]^ Interestingly, we saw highly variable CHOP expression even in litter mates. The up‐regulation of CHOP is not related to mouse age and activating transcription factor 6α (ATF6α) protein level in this model.

We are aware of the age‐related changes in the number of Z alpha‐1 antitrypsin globules found in the liver of murine models of alpha‐1 antitrypsin deficiency, and as such, we studied mice only between the ages of 6 and 12 months. In our murine models, we have noted a decrease in Z alpha‐1 antitrypsin globules while the alpha‐1 antitrypsin levels in plasma remain constant as the mice age. This observation leads us to believe that decreases in globules are caused by cellular adaptation to Z alpha‐1 antitrypsin disposal rather than down‐regulation of alpha‐1 antitrypsin gene expression. This hypothesis needs to be explored further.

We appreciate Professors Piccolo and Brunetti‐Pierri's recognition that M control mice open new avenues of study, and we are submitting both M and Z mice to the Jackson Laboratory so they will be available to other researchers.

## CONFLICT OF INTEREST

Nothing to report.
